# Pre-operative radiotherapy is associated with superior local relapse-free survival in advanced synovial sarcoma

**DOI:** 10.1007/s00432-022-04051-9

**Published:** 2022-06-10

**Authors:** Monika Scheer, Erika Hallmen, Christian Vokuhl, Jörg Fuchs, Per-Ulf Tunn, Marc Münter, Beate Timmermann, Sebastian Bauer, Anton George Henssen, Bernarda Kazanowska, Felix Niggli, Ruth Ladenstein, Gustaf Ljungman, Angelika Eggert, Thomas Klingebiel, Ewa Koscielniak, Cooperative Weichteilsarkom Studiengruppe [CWS]

**Affiliations:** 1 Department of Pediatric Hematology and Oncology, Charité –Universitätsmedizin Berlin, Corporate member of Freie Universität Berlin and Humboldt-Universität Zu Berlin, Augustenburger Platz 1, 13353 Berlin, Germany; 2 Pediatrics 5 (Oncology, Hematology, Immunology), Klinikum Stuttgart, Olgahospital, Stuttgart, Germany; 3 Pediatric Pathology, University Hospital of Bonn, Bonn, Germany; 4 Department of Pediatric Surgery and Urology, University Children’s Hospital, Tuebingen, Germany; 5 Department of Tumororthopedics, Helios-Klinikum, Berlin-Buch, Germany; 6 Department of Radiation Oncology, Klinikum Stuttgart, Stuttgart, Germany; 7 Department of Particle Therapy, University Hospital Essen, West German Proton Therapy Centre Essen (WPE), West German Cancer Center (WTZ), German Cancer Consortium (DKTK), Essen, Germany; 8 Sarcoma Center, West German Cancer Center, University of Duisburg‐Essen, Essen, Germany; 9 Department of Pediatric Oncology, University of Wroclaw, Wroclaw, Poland; 10 Department of Pediatric Oncology, University of Zuerich, Zuerich, Switzerland; 11 St. Anna Kinderspital and St. Anna Kinderkrebsforschung E.V, Vienna, Austria; 12 Pediatric Oncology, Department of Women’s and Children’s Health, Uppsala University, Uppsala, Sweden; 13 Hospital for Children and Adolescents, Goethe-University Frankfurt (Main), Frankfurt, Germany; 14 University of Tuebingen, Tuebingen, Germany

**Keywords:** Soft tissue sarcoma, Synovial sarcoma, Local therapy, Chemotherapy, Maintenance therapy, Radiotherapy, Scheduling of radiotherapy, Pre-operative radiotherapy, Surgery

## Abstract

**Purpose:**

Optimization of local therapies in synovial sarcoma (SS) considered unresectable at diagnosis is needed. We evaluated the effects of neoadjuvant versus adjuvant radiation versus surgery only on long-term outcomes.

**Methods:**

Patients with macroscopic SS tumors before chemotherapy (IRS-group-III) in the trials CWS-81, CWS-86, CWS-91, CWS-96, CWS-2002-P and SoTiSaR-registry were analyzed. Local therapies were scheduled after 3 neoadjuvant chemotherapy cycles.

**Results:**

Median age of 145 patients was 14.5 years. 106 survivors had median follow-up of 7.0 years. Tumor site was 96 extremities, 19 head–neck, 16 shoulder/hip, 14 trunk. Tumors were < 3 cm in 16, 3–5 cm in 28, 5–10 cm in 55, > 10 cm in 34 patients. In a secondary resection during chemotherapy, R0-status was accomplished in 82, R1 in 30, R2 in 21 (12 missing). Radiotherapy was administered to 115 (R0 61, R1 29, R2 20, missing 5), thereof 57 before and 52 after tumor resection. 23 were treated with surgery only. For all patients, 5 year event-free (EFS) and overall survival (OS) was 68.9% ± 7.6 (95%CI) and 79.1% ± 6.9. To establish independent significance, tumor site, size, surgical results and sequencing of local therapies were analyzed in a Cox regression analysis. Variables associated with EFS and OS are site, size and sequencing of local therapies. Variables associated with local recurrence are site, surgical results and sequencing of local therapies. The only variable associated with suffering metastatic recurrence is tumor size.

**Conclusion:**

Differences in sequencing of local therapy procedures are independently associated with outcomes. Best local control is achieved when tumors are irradiated pre-operatively and undergo R0 or R1 resection thereafter.

**Supplementary Information:**

The online version contains supplementary material available at 10.1007/s00432-022-04051-9.

## Introduction

Soft tissue sarcomas represent nearly 8% of childhood malignancies. Synovial sarcoma is the most common non-rhabdomyosarcoma soft tissue sarcoma (Goldblum et al. 2014; Pizzo et al. 2011). It typically affects the extremities of adolescents, as well as of young adults with main age range between 10 and 40 years (Goldblum et al. 2014; Pizzo et al. 2011). Local management in SS tumors considered unresectable at first diagnosis remains a major clinical challenge. The aim of every strategy remains long‐term survival, maximum control of local tumors while preserving function. Especially in children and adolescents with still growing tissue such as epiphyseal plates, potential late and long-term effects need to be considered. With improved multimodality therapy, widespread use of sparing surgery has become the foundation of curative‐intent surgical management. Radiotherapy has become an important component of a multimodality approach to advanced SS (Ferrari et al. 2015). However, the scheduling of radiotherapy delivery or rather the sequencing of local therapies remain a significant area of investigation. Beyond that, it is unclear whether or not patients in whom secondary complete resection with free margins succeeds during or after chemotherapy benefit from additional radiotherapy—despite initial failure to achieve respectability.

Therefore, our aim was not only to characterize variables and treatment procedures that may influence long-term survival, but also to clarify the potential effects of local therapy combinations. We aim to define the potential role of scheduling of radiotherapy delivery in a multimodal therapy approach if any. Our objective is to create a base for future therapy optimizations to improve the outcome for patients with initially unresectable SS disease.

## Materials and methods

Patients treated over the period of 1980–2013 in the consecutive trials CWS-81(Koscielniak, et al. 1992), CWS-86(Koscielniak et al. 1999), CWS-91(Dantonello et al. 2009), and CWS-96(Modritz, et al. 2005), CWS-2002-P(Koscielniak et al. 2013) and the registry CWS-SoTiSaR until 2013 were eligible if they fulfilled the following criteria: (i) SS diagnosis proven by central reference review, (ii) macroscopic tumor before the start of chemotherapy (classified as IRS III group), (iii) no evidence of metastases(Scheer et al. 2018), (iv) no previous treatment. All CWS-trials were prospective and approved by the appropriate ethics committees. Written informed consent was obtained from patients, guardians/parents or both.

Data collection was performed as previously described (Dantonello et al. 2009; Dantonello et al. 2008; Scheer 2021). Clinical information, treatment data, and outcome were available for all. Some had been included in previous analysis (pathological slides were reviewed for the purposes of those studies)(Stegmaier et al. 2017; Scheer et al. 2016a; Scheer et al. 2020; Scheer et al. 2019; Scheer et al. 2016b). The SYT–SSX fusion transcript was routinely analyzed since 2000. Disease was staged according to the Intergroup Rhabdomyosarcoma Study (IRS) post-surgical grouping system (Maurer et al. 1988). The IRS system categorizes patients in four groups based on the extent of residual tumor after initial surgery: group I includes completely excised tumors with negative microscopic margins, group II indicates grossly resected tumors with microscopic residual disease, group-III patients have macroscopic residual disease after incomplete resection or biopsy, and group-IV patients have metastases at onset (Maurer et al. 1988). Only IRS III group patients were included in this analysis.

According to the clinical tumor–node–metastases (TNM) classification (Harmer et al. 1970), T1 are tumors confined to the organ or tissue of origin, while T2 lesions invade contiguous structures. Regional node involvement is indicated as N0 or N1, based on histological or clinical/radiological assessments (Harmer et al. 1970).

## Treatment

The consecutive CWS protocols included specific therapy recommendations for SS. Disease was stratified by surgical stage (IRS) and nodal involvement. Treatment included a risk-stratified multimodal approach with recommendations for surgery, systemic chemotherapy, and radiotherapy.

## First surgery

According to the surgical guidelines of the respective CWS protocols biopsy should be the initial surgical procedure after imaging of primary tumor and regional lymph nodes—except when primary excision with adequate tumor-free margins (R0) and without functional impairment or mutilation was possible. Primary resection was indicated if there was no clear clinical evidence of lymph node involvement or metastatic disease, and if the tumor could be excised with adequate tumor-free margins (R0) and without functional impairment or mutilation. To achieve complete resection (R0) in patients with macroscopic or microscopic tumor residue (certain or doubtful) after primary biopsy or primary inadequate operation, a primary re-operation should be performed before any other therapy, if this can be done without mutilation or functional impairment. The interval between initial surgical intervention and chemotherapy including primary re-excision should not exceed four weeks. If a primary marginal excision or excisional biopsy (not recommended but often encountered as an initial situation) had already been done or if histological evaluation was inadequate, primary re-operation was to be considered. In general, this was the case in small, localized and well circumscribed tumors. The possibilities of reconstructive surgery were to be considered. In all other cases, chemotherapy was recommended to shrink and make the tumor more amenable to subsequent surgery.

## Chemotherapy

After initial surgery, the consecutive CWS protocols recommended chemotherapy in all SS patients. The chemotherapeutic regimens were VACA (vincristine, actinomycin-D, cyclophosphamide, and adriamycin) in CWS-81 (Koscielniak, et al. 1992), and VAIA-regimen, incorporating ifosfamide instead of cyclophosphamide, in CWS-86 (Koscielniak et al. 1999) and in all protocols including and following CWS-96 (Modritz, et al. 2005; Koscielniak et al. 2013). The CWS-91 trial evaluated therapy intensification with etoposide (EVAIA) (Dantonello et al. 2009). Since the CWS-96-protocol, the VAIA-regimen was used (Modritz, et al. 2005; Koscielniak et al. 2013). In all protocols, chemotherapy was risk stratified: 3 additional chemotherapy cycles were to be applicated for IRS III group SS patients (in contrast to IRS I and II group SS patients). In the CWS-2002P trial, the metronomic scheme cyclophosphamide/vinblastine was proposed after completion of intensive therapy. Due to the preference of the treating physician the oral metronomic scheme O-TIE (Klingebiel et al. 2008) was administered in some patients.

## Response evaluation

Response was assessed after three courses of neoadjuvant chemotherapy. For the purpose of this analysis, response assessment of the primary was based on the response evaluation criteria of the CWS for primary tumors and coded as follows: > 2/3 volume reduction, > 1/3 and < 2/3 volume reduction, < 1/3 or stable disease. Patients without response assessment included those with no documented response measurement, those evaluated at incorrect time points, and those who had a relevant tumor part removed during primary surgery.

## Best surgery

The surgical result of secondary tumor resection was categorized as the presence of a macroscopic [R2], or microscopic [R1] residual tumor or as a resection with free margins [R0] (Scheer et al. 2019).

## Radiotherapy

The consecutive CWS protocols had detailed radiotherapy recommendations. Radiotherapy was recommended for all SS IRS III group patients. According to the respective CWS-protocol radiotherapy at doses of 32–54.4 Gy (when accelerated hyperfractionated, 2 × 1,6 Gy/day) and 40–50 Gy if conventional fractionated dependent on response to chemotherapy (and resection status) was to be administered in analogy to recommendations for patients with rhabdomyosarcoma (Koscielniak, et al. 1992; Koscielniak, et al. 1999; Koscielniak, et al. 1994; Dantonello et al. 2009; Modritz, et al. 2005).

## Timing and scheduling of radiotherapy delivery

The respective CWS protocols foresaw a start of radiotherapy after obtaining response imaging after 3 cycles of chemotherapy (week 9) during weeks 9–12. Since the CWS-86 trial radiotherapy was recommended to be administered pre-operatively. In all CWS protocols, the application of radiotherapy was recommended in parallel with chemotherapy (continuation of chemotherapy without actinomycin-D or anthracyclines). According to the CWS-96 protocol all patients with SS (except for those who achieve R0 resection before chemotherapy, IRS group I) should be irradiated pre-operatively. A secondary R0 resection before radiotherapy was not recommended and should only be considered if radiation was impossible (and only in this way can omission of radiation be justified). According to the CWS-2002-P protocol patients with SS and a response of > 2/3 tumor volume reduction in week 9, in whom the conditions for a successful second-look surgery could be further improved by pre-operative radiation (e.g., by volume reduction of the residual tumor), were to be irradiated with 44.8 Gy pre-operatively. In patients with SS and a tumor response of < 2/3 > 1/3, the sequence of local therapies should be decided individually (pre-operative radiation or radical even mutilating resection). All patients with a secondary R1 resection, performed before irradiation on the assumption that an R0 resection was possible, should be irradiated post-operatively with 44.8 Gy. Similarly, patients with secondary R0 resection should be irradiated to 44.8 Gy post-operatively in the case of a non-pre-operative irradiation. A local small-volume boost up to 51.2 Gy was permissible. Patients with SS registered in the SoTiSaR will follow the specific SS recommendations of the CWS Guidance: Pre-operative radiotherapy is strongly recommended. SS patients with IRS group II or III tumors and SS patients with lymph node involvement should be irradiated with doses of 50.4 Gy or 54 Gy in 28 or 30 F, respectively (conventional fractionation). An optional boost of 5.4 Gy (conventional fractionation) is allowed in case of progressive disease or poor response if considered as feasible in terms of organs at risk, age, etc. Alternatively, hyperfractionated, accelerated radiotherapy with 44.8 Gy, 2 × 1.6 Gy/day may be used according to the former CWS recommendations.

## Statistical methods

Statistics were calculated using IBM SPSS^®^ 27 (Armonk, New York, U.S.). Event-free survival [EFS], overall survival [OS], local recurrence-free survival [LRFS] and metastases-free survival [MFS] were calculated using the Kaplan–Meier estimator (Kaplan and Meyer 1958). For OS, the time from diagnosis to death or last follow-up was calculated, for EFS to first relapse/progression, death or last follow-up, for LRFS the time to local disease recurrence (included combined), death or last follow-up, for MFS to first occurrence of metastases, death or last follow-up. In those cases where the type of relapse was not documented (unspecified relapse), no event for either LRFS or MFS was documented in the data base. Confidence intervals [CI] for the Kaplan–Meier estimator were computed using Greenwoods Formula (Greenwood 1926) and are stated at the 95% level. For comparison the log-rank test was used. Multivariate analysis of potential prognostic factors was conducted using Cox’s proportional hazards regression method. A stepwise variable selection procedure (combination of forward and backward selection techniques) was applied to the selected covariates. Hazard ratios (HRs) with 95% confidence intervals, calculated according to the Wald method, are reported.

## RESULTS

### Characteristics

A total of 330 consecutive patients with a diagnosis of localized SS made between 1981 and 2013 were identified. 145 (44%) had macroscopic residuals (IRS III) after first surgery and before the start of chemotherapy. Therefore, they were included in the analysis. Median age of those 145 selected patients with localized SS and macroscopic residuals after first surgery was 14.5 years (range 0.2–33.2). 27/145 patients were < 10 years and 9/145 patients > 21 years. The gender distribution was nearly equal (Table 1). Localization of the tumor was 96 extremities (66%), 19 head–neck (13%), 16 shoulder or hip (11%), 14 trunk (10%).

**Table 1:** Univariate analysis of characteristics and therapies in 145 patients with initially unresectable synovial sarcoma (IRS III)

	*N* (%)	5 yr EFS (95% CI)	*p* value	5 yr OS (95% CI)	*p* value	5 yr LRFS (95% CI)	*p* value	5 yr MFS (95% CI)	*p* value
All patients	145	68.9 ± 7.6		79.1 ± 6.9		89.6 ± 5.1		81.7 ± 6.5	
Studies									
CWS-81 CWS-86 CWS-91 CWS-96 CWS-2002P SoTiSaR	6 (4) 10 (7) 15 (10) 42 (29) 49 (34) 23 (16)	66.7 ± 37.6 34.3 ± 31.2 93.3 ± 12.5 58.1 ± 15.3 69.3 ± 12.9 86.5 ± 14.1	***0.001***	66.7 ± 37.6 33.8 ± 31.0 93.3 ± 12.6 75.1 ± 13.3 83.5 ± 10.4 90.9 ± 12.0	** < *0.001***	83.3 ± 29.8 88.9 ± 20.6 100 ± 0 83.8 ± 12.0 87.6 ± 9.4 100 ± 0	*0.140*	83.3 ± 29.8 57.1 ± 36.7 93.3 ± 12.5 81.7 ± 12.3 77.4 ± 11.8 91.1 ± 11.8	***0.040***
Gender									
Female Male	73 (50) 72 (50)	71.8 ± 10.6 66.1 ± 11.0	*0.800*	84.3 ± 8.4 73.6 ± 10.6	*0.223*	89.6 ± 7.3 89.5 ± 7.4	*0.796*	84.3 ± 4.3 73.6 ± 5.4	***0.026***
Age [years]									
≤ 10 10–21 ≥ 21	27 (19) 109 (75) 9 (6)	74.1 ± 16.5 70.9 ± 8.6 18.8 ± 31.0	***0.004***	81.5 ± 14.7 81.8 ± 7.5 30.0 ± 34.4	***0.010***	84.8 ± 13.7 91.2 ± 5.5 80.0 ± 35.1	*0.632*	84.0 ± 14.4 83.5 ± 7.3 50.0 ± 34.7	***0.014***
Site									
Extremities Head–neck Shoulder–hip Trunk	96 (66) 19 (13) 16 (11) 14 (10)	74.7 ± 8.8 77.9 ± 19.2 43.3 ± 26.1 42.9 ± 25.9	***0.006***	83.6 ± 7.6 83.5 ± 17.1 57.1 ± 7.1 64.3 ± 10.4	***0.030***	93.2 ± 5.5 83.5 ± 17.1 90.0 ± 18.6 77.9 ± 22.1	*0.342*	79.6 ± 8.2 88.5 ± 14.9 78.6 ± 21.6 91.7 ± 15.7	*0.462*
Size									
< 3 cm 3–5 cm 5–10 cm > 10 cm no information	16 (11) 28 (19) 55 (38) 34 (23) 12 (8)	93.8 ± 12.0 85.2 ± 13.3 71.6 ± 12.2 35.0 ± 16.1	** < *0.001***	93.8 ± 12.0 96.3 ± 7.1 82.4 ± 10.4 54.6 ± 17.1	** < *0.001***	100 ± 0 88.9 ± 11.8 90.0 ± 8.4 87.4 ± 11.6	*0.415*	93.8 ± 12.0 96.3 ± 7.1 76.7 ± 11.6 69.8 ± 16.9	***0.019***
Size (5 cm)									
< = 5 cm > 5 cm No information	49 (34) 93 (64) 3 (2)	87.4 ± 9.4 58.1 ± 10.2	** < *0.001***	91.7 ± 7.8 71.6 ± 9.4	***0.003***	91.5 ± 8.0 88.3 ± 6.9	*0.421*	93.7 ± 6.9 74.2 ± 9.4	***0.004***
T-Status									
T1 T2 TX	69 (48) 70 (48) 6 (4)	76.7 ± 10.0 57.9 ± 12.0	***0.019***	83.7 ± 8.8 72.5 ± 10.8	*0.250*	93.9 ± 5.7 84.2 ± 9.0	*0.056*	80.5 ± 9.4 80.9 ± 9.8	*0.988*
N-Status									
N0 N1 NX	126 (87) 11 (8) 8 (6)	73.9 ± 7.8 43.6 ± 30.4	***0.048***	82.5 ± 6.9 60.6 ± 20.2	*0.131*	89.8 ± 5.5 90.0 ± 18.6	*0.927*	84.1 ± 6.5 63.0 ± 34.7	*0.403*
Chemotherapy									
CEVAIE EVAIA VACA VAIA No information	3 (2) 14 (10) 6 (4) 120 (83) 2 (1)	100 ± 0 92.9 ± 13.5 66.7 ± 37.6 64.9 ± 8.6	*0.119*	100 ± 0 100 ± 0 66.7 ± 37.6 77.3 ± 7.6	*0.356*	100 ± 0 100 ± 0 83.3 ± 29.8 88.1 ± 6.1	*0.483*	100 ± 0 92.9 ± 13.3 83.3 ± 29.8 79.4 ± 7.4	*0.366*
Response									
> 2/3 > 1/3 and < 2/3 < 1/3 or stable No information	27 (19) 19 (13) 37 (26) 62 (43)	84.4 ± 14.1 94.7 ± 10.0 70.1 ± 15.7	*0.271*	73.2 ± 17.1 63.2 ± 21.8 64.2 ± 15.7	*0.649*	80.5 ± 15.3 94.4 ± 10.6 97.0 ± 5.9	*0.066*	92.0 ± 10.6 73.3 ± 19–8 77.4 ± 13.9	*0.298*
Metron. Therapy									
No Yes No information	90 (62) 46 (32) 9 (6)	69.4 ± 9.6 66.6 ± 13.9	*0.520*	77.8 ± 8.8 83.7 ± 11.0	*0.523*	89.1 ± 6.7 93.3 ± 7.3	*0.381*	83.3 ± 8.0 79.8 ± 11.8	*0.427*
Radiotherapy									
Yes No No information	115 (79) 23 (16) 7 (5)	71.8 ± 8.2 43.6 ± 21.2	***0.002***	81.9 ± 7.3 56.7 ± 21.6	***0.001***	91.6 ± 5.3 75.6 ± 18.6	***0.009***	83.8 ± 6.9 62.0 ± 22.3	***0.043***
Timing of Radioth									
No radiotherapy Adjuvant/after res Neo-adj./before res No information	23 (16) 52 (36) 57 (39) 13 (9)	43.6 ± 21.2 73.1 ± 12.2 69.5 ± 12.2	***0.008***	56.7 ± 21.6 82.4 ± 10.6 83.3 ± 10.0	***0.002***	75.6 ± 18.6 86.0 ± 9.6 98.0 ± 3.9	***0.008***	62.0 ± 22.3 86.2 ± 9.6 81.7 ± 10.2	*0.118*
Best surgery									
R0 R1 R2 No information	82 (57) 30 (21) 21 (14) 12 (8)	70.0 ± 10.0 69.0 ± 16.9 61.5 ± 21.0	*0.564*	83.3 ± 8.2 78.3 ± 15.6 70.8 ± 19.8	*0.599*	80.3 ± 9.0 82.5 ± 13.9 85.7 ± 14.9	*0.756*	80.3 ± 9.0 82.5 ± 13.9 85.7 ± 14.9	*0.980*
All patients	Median	EFS (range) 5.3 (0.1–17.6)		OS (range) 6.3 (0.5–18.1)		LRFS (range) 5.7 (0.5–17.6)		MFS (range) 5.7 (0.5–17.6)	

Bold values indicate statistical significance (p<0.05)

Tumors were < 3 cm in 16 (11%), 3–5 cm in 28 (19%), 5–10 cm in 55 (38%), and > 10 cm in 34 (23%) patients. For 10 tumors, size merely was documented as smaller or larger 5 cm, thereof 5 tumors > 5 cm and 5 tumors < 5 cm. In the remaining 3 patients, no size was documented. 69 (48%) tumors were documented as T1, 70 (48%) as T2 (6 tumors TX, 4%). For 11 (8%) affected lymph nodes were documented (5 pathologically proven).

### Chemotherapy

All 145 patients received neoadjuvant and adjuvant chemotherapy—conducted according to the respective protocol at the time of diagnosis. The majority of 120 (83%) patients received the VAIA-regimen (Vincristine, Actinomycin-D, Ifosfamide, Adriamycin), 14 (10%) EVAIA (Etoposide, Vincristine, Actinomycin-D, Ifosfamide, Adriamycin), 6 (4%) VACA (Vincristine, Actinomycin-D, Cyclophosphamide, Adriamycin), 3 patients received CEVAIE (Carboplatin, Epirubicine, Vincristine, Actinomycin-D, Ifosfamide, Etoposide) due to individual decision of the treating center. In the remaining 2, the conducted chemotherapy regimen was not documented. According to CWS protocols, chemotherapy was continued during radiotherapy with omission of Actinomycin-D, Adriamycin or Epirubicine. 46 (32%) patients received a maintenance therapy after completion of intensive chemotherapy.

### Response

Evaluation of response rates and chemotherapy is shown in Table 2.

**Table 2:** Multivariate analysis

Variables	EFS Hazard ratio	CI (95%)	*p*-value	OS Hazard ratio	CI (95%)	*p*-value	LRFS Hazard ratio	CI (95%)	*p*-value	MFS Hazard ratio	CI (95%)	*p*-value
Combination of local therapies												
Neo-adj/ before res	1		***0.010***	1		***0.004***	1		***0.001***	1		*0.408*
Adjuvant/ after res	0.669	0.290–1.548	*0.348*	0.995	0.364–2.718	*0.992*	163.227	5.864–4543.310	***0.003***	0.695	0.269–1.792	*0.452*
Surgery only	2.770	1.089–7.042	***0.032***	4.913	1.631–14.801	***0.005***	382.552	16.035–9126.587	** < *0.001***	1.431	0.489–4.187	*0.513*
Best surgery												
R0	1		*0.996*	1		*0.658*	1		***0.006***	1		*0.794*
R1	0.967	0.425–2.201	*0.936*	1.228	0.500–3.017	*0.655*	1.609	0.330–7.855	*0.557*	1.007	0.383–2.644	*0.989*
R2	1.006	0.378–2.682	*0.990*	1.682	0.539–5.252	*0.371*	90.073	5.623–1442.821	***0.001***	0.661	0.194–2.248	*0.508*
Site												
Extremities	1		***0.079***	1		*0.105*	1		*0.105*	1		*0.497*
Shoulder–hip	2.163	0.864–5.417	*0.100*	2.968	1.079–8.165	***0.035***	1.392	0.128–15.201	*0.786*	0.561	0.158–1.991	*0.371*
Head–neck	1.683	0.540–5.248	*0.370*	1.392	0.388–4.995	*0.612*	6.466	1.206–34.667	***0.029***	0.825	0.180–3.774	*0.804*
Trunk	3.154	1.263–7.880	***0.014***	2.997	1.042–8.623	***0.042***	4.001	0.767–20.865	*0.100*	0.247	0.032–1.898	*0.179*
Size												
< 5 cm	1		**< *0.001***	1		***0.026***	1		*0.284*	1		***0.006***
5–10 cm	2.989	1.073–8.327	*0.036*	3.547	0.996–12.628	*0.051*	0.626	0.124–3.149	*0.570*	5.436	1.542–19.169	***0.008***
> 10 cm	7.079	2.456–20.398	** < *0.001***	5.955	1.604–22.114	***0.008***	2.252	435–11.652	*0.333*	8.653	2.284–32.789	***0.001***

### Surgery

By tumor excision during chemotherapy (usually performed after 3 or 4 cycles as recommended in the CWS protocols), 82 of 145 (57%) patients achieved R0, 30 (21%) patients R1, and 21 (14%) R2-status as best surgical result. For 12 patients, information on resection status was not documented.

### Radiotherapy

Radiotherapy was conducted in 115 (79%) patients. 23 patients did not receive radiotherapy (no information documented for 7). Of those 115 irradiated patients 57 were irradiated before tumor excision (neoadjuvant) and 52 after tumor excision (adjuvant). The majority received < 50 Gy.

### Sequencing of local therapies according to tumor site

Among 96 tumors of the extremities, information about scheduling of radiotherapy was available for 86. Thereof 36 (38%) tumors were irradiated neoadjuvant, 31 (32%) adjuvant and 19 (20%) were not irradiated. Among 16 tumors of the shoulder or hip, 8 (50%) tumors were neoadjuvant, 7 (44%) adjuvant and 1 (6%) not irradiated. Among 14 SS tumors of the trunk, information was available for 13. Thereof 3 (23%) tumors were neoadjuvant, 9 (69%) adjuvant and 1 (8%) not irradiated. Among 19 tumors of the head-neck, information was available for 17. Thereof 10 (59%) tumors were irradiated pre-operatively, 5 (29%) post-operatively and 2 (12%) were not irradiated.

### Outcome

**Fig. 1:**
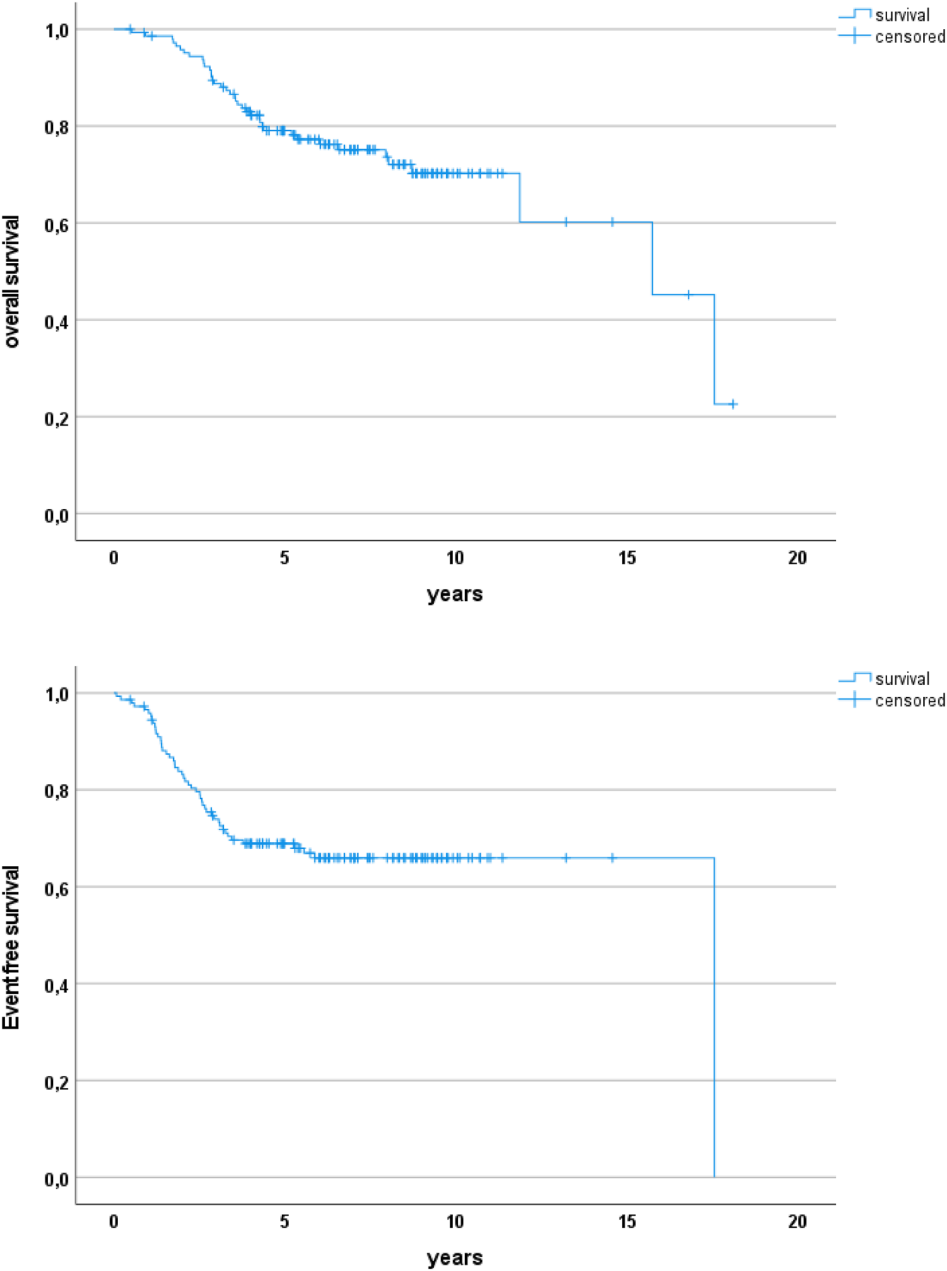
Overall and event-free survival of 145 patients with initially unresectable synovial sarcoma (IRS III)

3-, 5- and 10-year EFS probability was 74.0% ± 7.3 (95%CI), 68.9% ± 7.6 and 66.0% ± 8.0, respectively (Fig. 1); 3-, 5- and 10-year OS was 88.7% ± 5.3 (95%CI), 79.1 ± 6.9, and 70.2% ± 8.8. The 3-, 5- and 10-year LRFS was 93.5% ± 4.1 (95%CI), 89.6% ± 5.1 and 88.5% ± 5.5, respectively, and the 3-, 5- and 10-year MFS was 85.6% ± 5.9 (95%CI), 81.7% ± 6.5 and 73.2% ± 9.8.

140/145 (97%) patients achieved a first complete remission. 40 of those 140 suffered relapse, thereof 13 (9%) local, 23 (16%) metastatic and 1 (1%) combined. In 3 (2%) patients type of relapse was not specified.

100 (69%) patients remained in continuous first remission. With a median follow-up of 7.0 years (range 0.5–18.1) for survivors, 106 (73%) patients were alive at the cut-off date.

39 (27%) patients died. Cause of death was the disease in 31 patients, complications of therapy in 3, a second malignancy in 1, and not documented in 4 patients. Median time to local failure was 2.9 years. The latest local recurrences were documented at 3.3 and 5.3 years, respectively.

Median time to distant failure without involvement of the primary region was 2.3 years. The latest occurrence of metastases was documented at 3.8, 5.6 and 5.9 years.

### Factors for survival

In the univariate analysis, the conducted CWS studies were associated with EFS, OS and MFS (Table 1).

Factors associated with adverse EFS were older patients’ age ≥ 21 years, tumor located at the shoulder or hip or else in the trunk, large and very large tumor size (5-10 cm, > 10 cm), invasive tumor growth pattern (T2), positive lymph node involvement (N1) and no conduction of radiotherapy (Table 1). Factors associated with adverse OS were older patients’ age > 21 years, tumor located at the shoulder or hip or else in the trunk, large or very large tumor size (5–10 cm, > 10 cm), and no conduction of radiotherapy.

### Factors for local and metastatic relapse

**Fig. 2:**
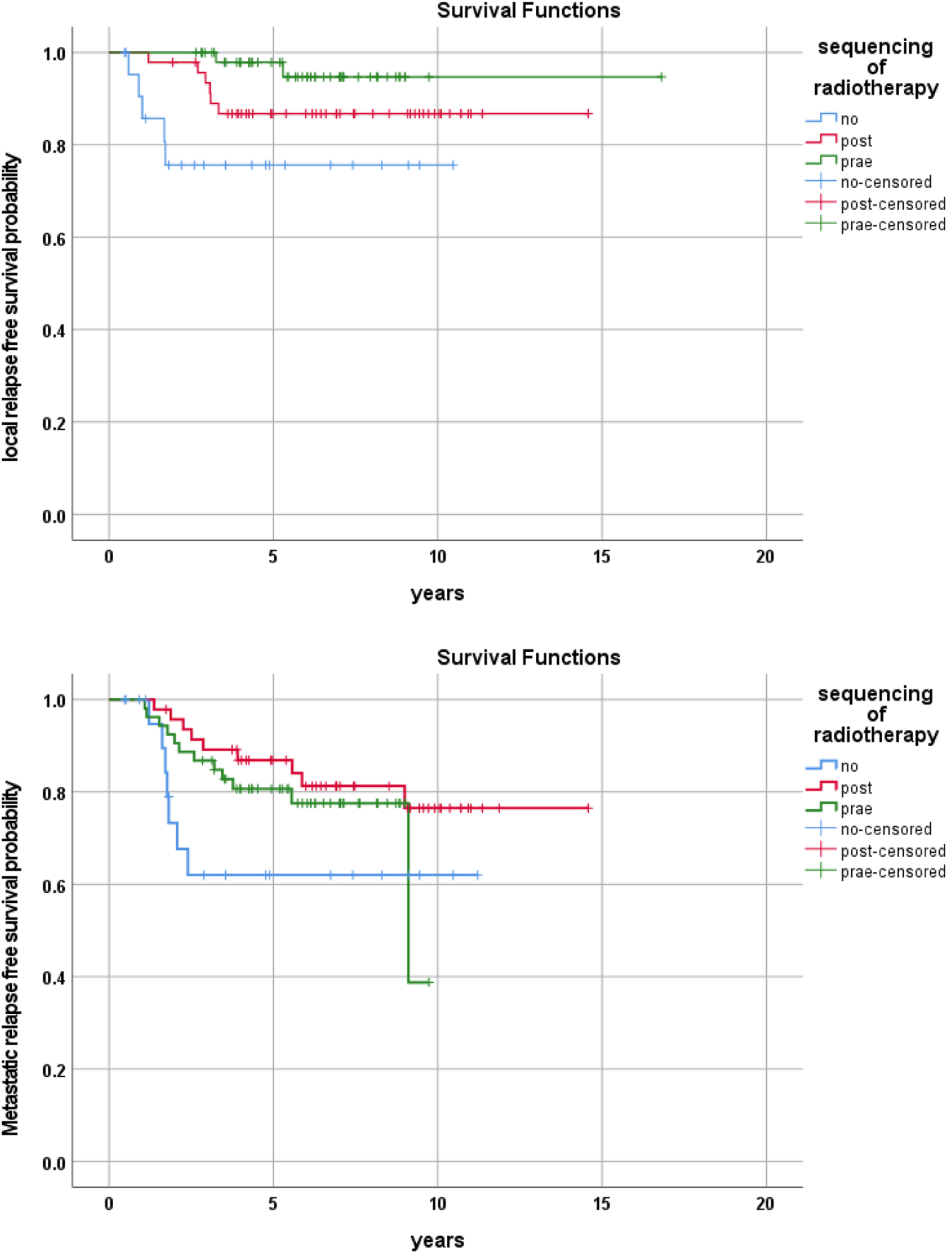
Local relapse-free and metastatic relapse-free survival probabilities according to scheduling of radiotherapy

In the detailed evaluation of local disease recurrences (Table 1, Fig. 2), the conduction of radiotherapy was the only factor which correlated with LRFS in univariate analysis. Patients who underwent radiotherapy before tumor resection had a local relapse-free survival probability of 98.0 ± 3.9% (*p* = *0.008*).

Older patients’ age (≥ 21 years), male gender, large and very large tumor size (5–10 cm, > 10 cm), and no conduction of radiotherapy correlated with adverse metastatic free survival probability.

### Cox regression analysis

**Fig. 3:**
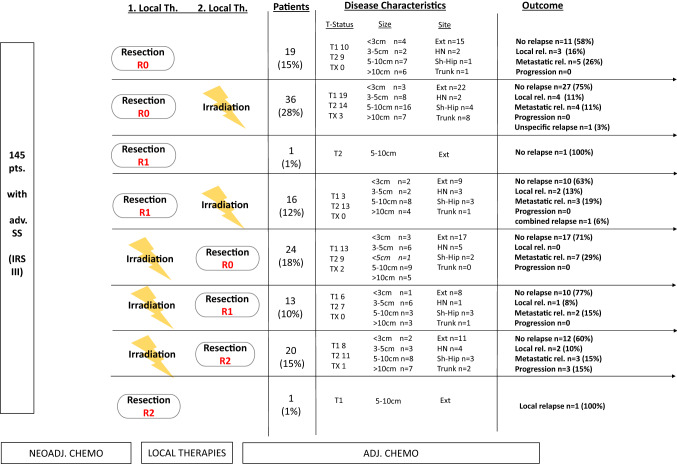
Sequencing of local therapies, disease characteristics and outcome of treated patients

To establish independent prognostic significance of tumor site, size, surgical result and combination/sequencing of local therapies, these variables were included in the multivariate model (Table 3, Fig. 3).

**Table 3:** Conducted chemotherapies and responses

Chemotherapy	Patients	> 2/3 volume reduction	> 1/3 and < 2/3 volume reduction	< 1/3 reduction or stable disease	No information
CEVAIE	3	0	0	1	2
EVAIA	14	1	1	2	10
VAIA	120	23	18	33	46
VACA	6	3	0	1	2
No information	2	0	0	0	2

Tumor site, size and combination/sequencing of local therapies were independently associated with EFS and OS. Variables which were independently associated with suffering a local relapse are tumor site, surgical result and combination/sequencing of local therapies. The only variable independently associated with suffering a metastatic relapse was tumor size.

When including only SS tumors > 5 cm in the multivariable model significance of results did not differ (supplementary table 1).

### Chemotherapy and response evaluation

When correlated with survival probabilities, there was no superiority of a particular intensive chemotherapeutic regimen (Table 1). Tumor response to chemotherapy did not correlate with EFS, OS and MFS; whereas, the local relapse-free survival probability was of borderline significance, *p* = *0.066*. Paradoxically, tumors with a volume decrease of more than 66% seemed to have a higher risk of local recurrence than tumors, which react with less volume decrease or remain stable.

The application of maintenance therapy after completion of intensive multimodal therapy did not correlate with better survival probabilities.

### Patients with secondary R0 resection during chemotherapy treated without radiotherapy

Nineteen patients achieved a secondary complete resection (R0) while on chemotherapy and did not receive a subsequent irradiation.

Among those 19 non-irradiated patients with secondary R0 resection, 3 local relapses were documented. Eight of those 19 patients had undergone amputation. Among these 8 mutilated patients 5 remained in first complete remission, none suffered a local relapse (0%) and 3 suffered metastatic relapse. Among those other 11 non-mutilated patients 6 remained in first complete remission, 3 (27%) suffered local relapse, 2 suffered metastatic relapse.

### Patients with R1 and R2 resection status after neoadjuvant radiotherapy

13 patients underwent R1 resection after neoadjuvant irradiation (Fig. 3). Merely one of those 13 patients (8%) suffered local relapse. 2 other patients (15%) suffered metastatic relapse. Overall, 10/13 patients (77%) remained disease free.

Among 20 patients who underwent R2 resection after neoadjuvant irradiation, 2 suffered local relapse (10%), 3 suffered metastatic relapse (15%), 3 suffered disease progression (15%). Overall, 60% remained disease free.

### Patients with lymph node involvement

11 patients were reported with lymph node involvement at first diagnosis, thereof 5 pathological confirmed. For 3 patients resection of lymph nodes was reported, for 3 irradiation of the affected lymph nodes. Among those 11 patients none suffered isolated lymph node relapse. 1 suffered local relapse, 2 suffered metastatic relapse, 1 unspecific relapse. For 2 patients disease progression with therapy was reported. Overall, 5/11 (45%) died of their disease.

## Discussion

Synovial sarcomas are rare tumors with 800 new cases per a year in the USA (Pizzo et al. 2011) and have been explored in depth since the first article written by Haagensen and Stout in 1944 (Haagensen and Stout 1944). Synovial sarcoma is the most common non-rhabdomyosarcoma soft tissue sarcoma (Goldblum et al. 2014; Pizzo et al. 2011). Two very recent pediatric non-rhabdomyosarcoma soft tissue sarcoma clinical trials in Europe (European Pediatric Soft Tissue Sarcoma Study Group [EpSSG] NRSTS 2005) and North America (Children’s Oncology Group [COG] ARST0332) have validated presumed risk factors and thus optimized systemic therapy (Ferrari et al. 2017; Ferrari et al. 2021; Venkatramani et al. 2021; Weiss and Spunt 2021; Martin-Broto 2020). A major distinction between the two protocols is that the use and timing of radiotherapy were not mandated by the protocol in NRSTS 2005 as in ARST0332. A substantially higher rate of local relapses in NRSTS 2005 than in ARST03328 underlines the importance of local therapies and a standardized approach to local tumor control. In that respect, when compared to the great international efforts made to answer questions about SS risk factors and systemic therapies, very little attention has been paid to the optimization of local therapies. Especially for those tumors considered unresectable at diagnosis, there has been no change or further development in local therapies for decades. Whereas the benefit of radiotherapy in advanced SS is well documented (Venkatramani et al. 2021; Ferrari et al. 2021; Ferrari et al. 2004; Okcu et al. 2001; Okcu et al. 2003; Fuchs et al. 2021) the prognostic impact of  radiotherapy scheduling within the multimodal approach has never been investigated in depth and still needs to be clarified. The strength of our study is the large number of patients included, and all of whom were enrolled in prospective trials and treated according to specific recommendations for SS with regard to systemic and local therapies. All 145 patients analyzed here were treated in prospective risk-adapted trials with median follow-up of more than 7 years for survivors. A confirmation of diagnosis by central review was mandatory. In addition, retrospective pathological reevaluation of all available tissue had been performed for purpose of recent investigations (Stegmaier et al. 2017; Scheer et al. 2016a; Scheer et al. 2016b).

We can conclude that children, adolescents and young adults with initially unresectable SS treated according to CWS recommendations have a good prognosis with an expected 5 year and 10 year OS of 80% and 70%, respectively. With regard to first relapse patients had a local recurrence rate of 9%, a distant metastases rate of 16% and a combined relapse rate of both local and distant lesions of 1% (type of relapse not specified in 2%).

In our cohort, predictors of survival are mostly in accordance with literature. Nevertheless, we can add interesting data. Age remains a relevant factor with a significantly better outcome of those patients younger than 21 years. Patients older than 21 years have a significantly higher risk of suffering a metastatic relapse, despite of treatment according to pediatric protocols. There was no significant age-dependent difference in survival in the group of patients younger than 21 years. Patients with male gender are at higher risk for adverse metastatic events compared to females. Patients with tumors located at the trunk or as a new finding at the shoulder or hip had a worse EFS and OS compared to those with tumors at the extremities or located at the head/neck. According to pediatric soft tissue sarcoma protocols, sarcoma located at the shoulder or hip are categorized within the tumor site “extremity”. For this analysis which focused specifically on local therapies, they were evaluated as a separate group. We could clarify that outcome of SS located at the shoulder or hip when considered primary unresectable does not differ from those SS tumors located at the trunk (which was repeatedly confirmed as significantly worse in many studies). In contrast, outcome of grossly resected SS located at the shoulder or hip outcome does not differ from those located at the extremities (Scheer 2021). One interpretation is that this might reflect the complexity of local therapy of tumors at shoulders and hips in the advanced disease stage. However, this result might be noteworthy with regard to future risk stratifications, treatment recommendations and trials.

Larger tumor size was associated with worse survival. Patients with larger tumors were at independent risk for suffering metastatic recurrence. Though lymph node involvement is very rare in SS, in our series 11 patients were documented with affected lymph nodes, 5 with pathological confirmation. Those patients with affected lymph nodes had a significantly worse survival in terms of relapse and disease progression. Interestingly, not a single isolated lymph node relapse was reported. However, lymph node involvement seems to indicate a more aggressive biology—suggesting that despite its rarity not only a careful staging is necessary but also a great care during and after therapy for respective patients.

All analyzed patients received chemotherapy. No differences in survival between the intensive chemotherapeutic regimens could be identified. Recently, a benefit of adding maintenance therapy after completing intensive therapy was shown for high-risk rhabdomyosarcoma (Bisogno et al. 2019; Koscielniak and Klingebiel 2020). In this retrospective evaluation of 145 prospectively treated patients with unresectable SS at first diagnosis, the addition of maintenance therapy after completion of intensive multimodal therapy was not associated with improved survival probability. Most patients have received Cyc/Vbl. However, this finding underlines the need for subtype analyses in sarcoma basket trials.

According to the consecutive CWS protocols, the best local treatment approach was to be performed after the assessment of chemotherapy response obtained after 3 cycles of chemotherapy. The benefit of additional radiotherapy to patients in whom secondary R0 resection succeeds after 3 or 4 cycles of chemotherapy, despite initial failure to achieve resectability, could not be defined so far. It is still unclear whether the use of radiotherapy for these patients results in improved survival (Ferrari et al. 2015; Ferrari et al. 2004). Moreover, radiotherapy does not result in superior survival after primary R0 resection before the start of chemotherapy.

In this series, omission of radiotherapy was associated with worse EFS, OS and LRFS for all 145 analyzed SS patients. For those 19 patients with secondary R0 resection treated without subsequent radiotherapy, the local relapse rate of 16% was only slightly elevated when compared to those patients to whom post-operative radiotherapy was added after secondary R0 resection (local relapse rate of 11%). However, 8 of those 19 patients had undergone amputation. Radiotherapy had been omitted due to the fact, that the affected body part had been completely removed. Among those 11 other patients who underwent non-mutilating R0 resection without subsequent irradiation, 3 suffered local relapse. This results in a local recurrence rate of 27% for non-mutilated patients. Although the numbers are small, the risk of suffering local recurrence appears to be considerably increased when radiotherapy is omitted and the affected body part is not completely removed. In absolute contrast and even more interesting, not one single local relapse was documented for those patients who had undergone pre-operative radiotherapy and a subsequent R0 resection.

With regard to scheduling of radiotherapy the consecutive CWS therapy protocols recommended pre-operative radiotherapy (after 3 neoadjuvant chemotherapy cycles and response assessment). Pre-operative radiotherapy has potential advantages over post-operative radiotherapy: pre-operative irradiation can improve the chance to perform a complete secondary resection, may reduce the risk of intra-operative contamination and could use smaller radiotherapy fields. Moreover, the accuracy in defining the radiotherapy field is improved because the intact tumor target volume is easier to define; the residual tumor may act as a form of ‘spacer’, meaning that less uninvolved normal tissue is exposed to the higher radiotherapy dose; a significant proportion of the irradiated tissue will be removed surgically, which may reduce the risk of second tumors. Especially in pediatric patients this aspect seems of utmost importance. There is a biological rationale as the tumor and surrounding tissues are less hypoxic than in the post-operative setting and hypoxia increases tumor radio-resistance (Barker et al. 2015). In adult soft tissue sarcoma, pre-operative radiotherapy has been increasingly used in standard clinical settings. O'Sullivan (O'Sullivan et al. 2002) showed a small significant improvement in OS in adult patients with extremity STS randomized to receive pre-operative radiotherapy at 50 Gy instead of post-operative radiotherapy at 66 Gy, although this was counterbalanced by an increased risk of acute wound complications. Though pre-operative radiotherapy is being investigated in a number of non-rhabdomyosarcoma STS studies (e.g., NCT01344018 and NCT02180867), there is no published experience on scheduling of radiotherapy for SS so fare.

In this series, neoadjuvant radiotherapy is associated with a significantly lower risk of suffering local relapse independent of tumor site, size and resection status.

Nevertheless, treating physicians normally have major concerns to deliver pre-operative radiotherapy when a subsequent tumor resection may only be done with positive margins. However, our data do not show a higher rate of recurrences for those patients (Fig. 3).

Recent investigations have shown that initially adequately resected SS < 5 cm (IRS I), regardless of grade can be safely treated with a surgery only approach (Scheer 2021; Ferrari et al. 2017; Venkatramani et al. 2021)—consequently, they are now defined as low-risk SS. In this series of 145 IRS-III-group patients, 49 (34%) tumors were < 5 cm.

All consecutive therapy protocols had explicitly recommended primary resection for all tumors when primary excision with adequate tumor-free margins (R0) and without functional impairment or mutilation was considered possible. Therefore, it must be assumed that all analyzed tumors in this series were considered initially unresectable by the participating centers and did not meet the criteria for the nowadays so-called low-risk SS. However, in most cases this decision was not made centrally but by the respective treating centers. Since this decision strongly depends on surgical experience and expertise, it cannot be excluded that individual centers might have had a different estimation depending on their experience and medical focus. Thus, a potential heterogeneity within our series cannot be excluded. To get as close as possible to a valid statement, multivariate analysis for tumors > 5 cm only was performed. The independent relevance of the scheduling of radiotherapy was confirmed (supplementary table 1).

An inherent weakness of our study is that wound complications were not recorded. However, information on late effects was documented. The importance of late effects could exceed that of acute wound complications.

The result of this study might be compromised by a relatively small number of merely 145 patients. In contrast to all other evaluations on sequencing of local therapies in STS, this study is subtype-specific and not tumor site specific. Possibly too minor but not to be overlooked is the fact that, pediatric protocols recommend local therapy only after 3 neoadjuvant chemotherapy cycles and that chemotherapy is to be continued during radiotherapy only with omission of anthracyclines.

The resectability of a tumor depends critically on its localization, as do the expected late sequelae with different treatment modalities. Therefore, the choice of sequence of local therapy critically depends on the tumor site. In this series, 50% of the tumors located at the head-neck and at the shoulder or hip were pre-operatively irradiated, while only 38% of the extremity tumors and 21% of tumors located at the trunk received pre-operative irradiation. However, the independent significance from tumor site was shown in the cox regression analysis.

In the whole series, only 57 patients (39%) received their radiotherapy pre-operatively. Despite detailed recommendations on the scheduling of local therapies in the respective CWS protocols, these were not followed in a striking number of cases in the multinational and multicenter setting. In addition to study protocols, the CWS study center provided the option of a centralized interdisciplinary reference assessment with an individualized protocol-conform treatment recommendation for the respective patient, if desired by the treating physician, but not obligatory. The final treatment decision was made by the respective treating centers. Intra-institutional  might not paradigms have been amenable to protocol-compliant local therapy. Thus, again a potential heterogeneity within the analyzed series  cannot be excluded. One could argue that this potential heterogeneity might make meaningful comparisons challenging, but more likely it seems to reflect a clear real-life scenario in the conduction of multicenter and multinational trials. Moreover, it anticipates the pitfalls of future clinical trials, in which local therapies can no longer be ignored.

In summary, patients with initially unresectable SS treated according to CWS recommendations have a good prognosis and compare with those of other series (Venkatramani et al. 2021; Ferrari et al. 2021). To our knowledge this analysis represents the largest series of initially unresectable SS treated in prospective risk-adapted trials so far.

It is the first try of analyzing the prognostic relevance of scheduling the radiotherapy NRSTS-subtype specific and not site specific.

We could show that:

1. Sequencing of local therapies is of independent prognostic relevance in initially unresectable SS.
2. Pre-operative radiotherapy is associated with a lower risk of suffering local relapse independent of tumor site, size and resection status.
3. Omission of radiotherapy in initially unresectable SS results in a significant deterioration of outcome.
4. For patients who achieve a secondary complete resection with free margins, omission of radiotherapy results in a high rate of local recurrences for those patients who do not undergo amputation of the affected body part.
5. The survival of patients with initially unresectable SS located at the shoulder or hip does not differ from those suffering of SS located at the trunk. It is significantly worse compared to extremities suggesting the need for an adjusted risk stratification in future trials.

Finally, we can conclude that the prognostic relevance of the sequencing of local therapies and scheduling of radiotherapy delivery is highly underestimated—not only in initially unresectable SS but in the whole group of sarcoma diseases. Sequencing of local therapies urgently needs further investigations with high demand of sarcoma subtype-specific investigations.

## Supplementary Information

Below is the link to the electronic supplementary material.

Supplementary file1 (DOCX 25 kb)
