# Supplementary material for: Pre-operative radiotherapy is associated with superior local relapse-free survival in advanced synovial sarcoma
Source: J Cancer Res Clin Oncol. 2022 Jun 10;149(5):1717–31. doi: 10.1007/s00432-022-04051-9 (PMC10097790; doi:10.1007/s00432-022-04051-9)
Supplement: Supplementary file 1 — Supplementary file1 (DOCX 25 kb) [file 432_2022_4051_MOESM1_ESM.docx]

| **Variables** | **EFS**  Hazard ratio | CI (95%) | *p*-value | **OS**  Hazard ratio | CI (95%) | *p*-value | **LRFS**  Hazard ratio | CI (95%) | *p*-value | **MFS**  Hazard ratio | CI (95%) | *p*-value |
| --- | --- | --- | --- | --- | --- | --- | --- | --- | --- | --- | --- | --- |
| **Combination of local therapies**  neo-adj/ before res.  adjuvant/ after res.  surgery only | 1  0.496  2.092 | 0.206-1.193  0.773-5.659 | ***0.020***  *0.117*  *0.146* | 1  0.771  4.528 | 0.272-2.191  1.420-14.433 | ***0.005***  *0.626*  ***0.011*** | 1  523.441  978.078 | 8.058-34001.777  19.174-49893.662 | ***0.003***  ***0.003***  ***0.001*** | 1  0.480  1.171 | 0.172-1.342  0.375-3.658 | *0.248*  *0.162*  *0.786* |
| **Best surgery**  R0  R1  R2 | 1  1.046  0.765 | 0.442-2.474  0.268-2.183 | *0.846*  *0.918*  *0.617* | 1  1.383  1.612 | 0.550-3.475  0.506-5.143 | *0.654*  *0.491*  *0.419* | 1  2.042  182.110 | 0.339-12.315  6.343-5228.495 | ***0.008***  ***0.436***  ***0.002*** | 1  1.255  0.611 | 0.458-3.437  0.175-2.133 | *0.607*  *0.659*  *0.440* |
| **Site**  Extremities  Shoulder-Hip  Head-neck  Trunk | 1  2.118  2.035  3.522 | 0.824-5.441  0.533-7.769  1.369-9.063 | *0.065*  *0.119*  *0.298*  ***0.009*** | 1  3.148  1.780  3.383 | 1.113-8.907  0.477-6.641  1.144-10.000 | ***0.081***  ***0.031***  ***0.391***  ***0.028*** | 1  *1.224*  *5.572*  *4.904* | 0.101-14.763  0.607-51.148  0.825-29.149 | *0.192*  *0.874*  *0.129*  *0.080* | 1  *0.548*  *1.212*  *0.266* | 0.152-1.979  0.246-5.972  0.034-2.071 | *0.500*  *0.358*  *0.813*  *0.206* |
| **Size**  5-10cm  >10cm | 1  2.398 | 1.199-4.794 | ***0.013*** | 1  1.636 | 0.741-3.611 | *0.223* | 1  3.762 | 0.710-19.930 | *0.119* | 1  1.685 | 0.723-3.928 | *0.227* |
